# Fabrication of a Multi-Walled Nanotube (MWNT) Ionic Liquid Electrode and Its Application for Sensing Phenolics in Red Wines

**DOI:** 10.3390/s90906701

**Published:** 2009-08-26

**Authors:** Kyo-Il Kim, Hee-Young Kang, Jae-Chan Lee, Seong-Ho Choi

**Affiliations:** Department of Chemistry, Hannam University, Daejeon 305-811, Republic of Korea; E-Mails: sinjangkki@naver.com (K.K); dartpip@nate.com (H.K); jclee@hnu.kr (J.L)

**Keywords:** MWNT ion liquid electrode, glycidyl methacrylate, radiation-induced graft polymerization, phenolics, red wines

## Abstract

A multi-walled nanotube (MWNT) ionic liquid was prepared by the immobilization of 1-butylimidazole bromide onto an epoxy group on a poly(glycidyl methacrylate)-grafted MWNT, which was synthesized by radiation-induced graft polymerization of glycidyl methacrylate onto MWNT in an aqueous solution. Subsequently, a MWNT ionic liquid electrode was fabricated by hand-casting MWNT ionic liquid, tyrosinase, and chitosan solution as a binder on indium tin oxide (ITO) glass. The sensing ranges of the MWNT ionic liquid electrode with immobilized tyrosinase was in the range of 0.01-0.08 mM in a phosphate buffer solution. The optimal conditions such as pH, temperature, and effects of different phenolic compounds were determined. The total phenolic compounds of three commercial red wines were also determined on the tyrosinase-immobilized biosensor.

## 1. Introduction

A great number of papers on carbon nanotube (CNT)-based sensors have been published over the past several years mainly because CNTs have the following advantages for electrochemical sensor applications: (1) small size with a large surface area, (2) high sensitivity, (3) fast response time, (4) enhanced electron transfer and (5) easy protein immobilization on CNT-modified electrodes, coupled with the fact that several methods have been developed [1–3].

In recent years, direct electrochemistry of biologically important enzymes has been studied with ionic liquids in both theoretical and practical applications because ionic liquids, with their high polarity, non-coordination power, high selectivity, fast rates and great enzyme stability are considered to be suitable media for supporting biocatalytic processes [4–5]. Enzymes are usually active and protein refolding is improved in ionic liquids [6]. Zeng and coworkers fabricated a modified GC electrode by entrapping glucose oxidase (GOx) in a nano Au particle-ionic liquid-*N,N*-dimethylformamide composite film [7]. Sun *et al*. published information about the fabrication of a modified imidazolium-based carbon ionic liquid electrode by entrapping Hb into a sodium alginate hydrogel film [8]. Safavi *et al*. constructed a modified pyridium-based carbon ionic liquid electrode using octylpyridinium chloride ([OcPy][Cl]) to immobilize direct hemoglobin (Hb) on carbon ionic liquid electrode (CILE) [9]. However, to the best of our knowledge, no papers on CNT ion liquid electrodes for the immobilization of enzymes have been published until now, because it is very difficult for the ion liquids to be introduced onto a CNT surface.

Radiation-induced graft polymerization (RIGP) is a useful method for the introduction of functional groups into different polymer matrixes using specially selected monomers. There have been several reports on radiation-induced graft polymerization of polar monomers onto polymer substrates with hydrophobic properties to obtain hydrophilic properties for versatile applications [10–11]. The RIGP method can be easily modified for the surface of MWNTs. In a previous paper [13], a MWNT was modified by various vinyl monomers in an aqueous solution at room temperature by RIGP. In particular, the epoxy groups of poly(glycidyl methacrylate) [poly(GMA)] are changeable to alcohols [14], amines [15], phosphonic acids [16], sulfonic acids [17], etc [18]. However, little has been reported about the introduction of ionic liquids onto an epoxy group of a grafted poly(GMA).

Wines, particularly red wines, contain numerous biologically active compounds, the most important of which are polyphenols. The nutritional importance of polyphenols is attributed to their antioxidant properties and there has been increasing interest in the flavonoids and related phenolics which are naturally found in red wines [19]. Red wines have been reported to be preventive of many ailments and may possibly play a role in reducing thrombotic and anthrogenic processes. In addition, polyphenols also contribute substantially to the quality of wines, affecting their color, flavor, stability and aging behavior [20]. However, little has been reported regarding the easy determination of the total amount of phenolics in red wine by an electrochemical method.

In this study, we synthesized poly(GMA)-grafted MWNT by RIGP of GMA in an aqueous solution in order to immobilize imidazole bromide as an ionic liquid. A MWNT ionic liquid electrode was prepared on an ITO electrode by hand casting of MWNT ionic liquid, tyrosinase, and chitosan as a binder. The sensing efficiency of the prepared MWNT ionic liquid electrode for phenol was evaluated in a phosphate buffer solution. The optimal conditions such as pH, temperature, and effects of different phenolic compounds were evaluated. Total phenolic compounds for three commercial red wines were also analysed using the prepared enzyme electrode.

## 2. Experimental

### 2.1. Reagents

Tyrosinase from mushrooms (EC 1.14.18.1), phenol, *p*-cresol, catechol, glycidyl methacrylate and chitosan were purchased from the Sigma-Aldrich Korea Ltd. Company. An ITO electrode as a working electrode (working area 0.7 × 1.1 cm^2^, 10 Ω resistance) was purchased from Dasom RMS Co. (Korea). MWNT (CM-95) was supplied by Hanwha Nanotech Co., Ltd (Korea). Three Korean red wines (Chateau Mani-dry, Chateau Mani-sweet, and Chateau Mani-nouveau) were used in order to provide phenolics. Solutions for the experiments were prepared with water purified in a Milli-Q puls water purification system (Millipore Co. Ltd.; the final resistance of water was 18.2 MΩcm^−1^) and it was degassed prior to each measurement.

### 2.2. Synthesis of the poly(GMA)-g-MWNT

The MWNT’s were purified in order to remove the catalyst and non-crystallized carbon impurities. Briefly, MWNT’s were treated with a 3:1 (vol–%) mixture of H_2_SO_4_/HNO_3_ and in the process MWNT’s were cut into shorter segments. The purified and cut MWNT were used as the supporting materials for grafting with GMA. The MWNT (2.0 g) and GMA (2.0 g) were mixed in an aqueous solution (20 mL). Nitrogen gas was bubbled through the solution for 30 minutes to remove oxygen gas, and the solution was irradiated by γ-rays from Co-60 source under atmospheric pressure and ambient temperature. A total irradiation dose of 30 kGy (a dose rate = 1.0 × 10^4^ Gy/h) was used. The poly(GMA)-*g*-MWNT’s were dried in a vacuum oven at 50 °C for 8 hrs.

### 2.3. Fabrication of the Enzyme Electrode Based on Carboxylic Acid-modified MWNT

**Scheme 1.**
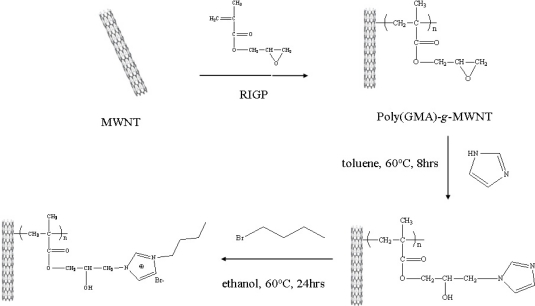
Synthesis procedure of the MWNT ionic liquid for biosensors.

Scheme 1 shows the fabrication procedure of the MWNT ionic liquid electrode based on an epoxy group-modified MWNT prepared by γ-irradiation. A mixed solution containing chitosan (3.2 mg) as a binder, tyrosinase (25,000 units, 0.3 mg), and MWNT ionic liquid (3.2 mg) as the supporter was prepared in an acetic acid solution (0.01 mL), and then the mixed solution (9.0 μL) was coated on the surface of a pre-cleaned ITO electrode by the hand-cast method. The prepared MWNT ion liquid electrode was kept at 4 °C until used.

### 2.4. Instrumentation

Cyclic voltammetric (CV) experiments were performed with a Potentiostat/Gavanostat model 283 (Ametek PAR, U.S.A.) or CV-50w voltametry (Bioanalytical Systems, Inc. U.S.A.). All experiments were carried out with a conventional three-electrode system. The working electrode was an ITO electrode coated with the poly(GMA)-g-MWNT, counter electrode was the platinum wire, and reference electrode was an Ag/AgCl (sat’d KCl). The surface morphology of the samples was determined using scanning electron microscopy (SEM, S-3000N, Hitachi Science System Ltd., Japan), and by HR-TEM (JEOL, JEM-2010, USA). The thermal gravimetric analysis (TGA) was conducted on a Scinco TGA S-1000 (Seoul, Korea) under N_2_ flow from 25 °C to 700 °C at a heating rate of 20 °C/min.

## 3. Results and Discussion

### 3.1. Fabrication and Characterization of MWNT Ion Liquid Electrode

The functionalization of MWNT is one of the most active fields in nanotubes research and it is an effective tool to broaden the electrochemical application spectrum of MWNT. We functionalized MWNT from a GMA monomer in the presence of MWNT by RIGP in an aqueous solution, in order to introduce the epoxy group. The epoxy group can be converted into an ionic liquid group such as imidazolium cations for the purpose of enhancing electron transfer. In our previous paper, the morphology, physical and chemical properties for the vinyl polymer-grafted MWNT obtained by RIGP were evaluated [13]. In order to enhance electron transfer, we introduced butyl imidazolium bromide according to Scheme 1 and carried out the corresponding elemental analysis (Table 1).

The content of imidazolium ion was estimated to be approximately 11.0% by the elemental analysis. This indicates that butyl imidazolium cations were successfully introduced onto an epoxy group of poly(GMA)-g-MWNT.

**Figure 1.**
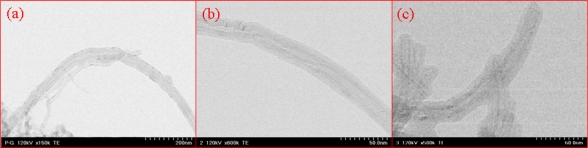
TEM images of pure MWNT (a), poly(GMA)-g-MWNT (b) and MWNT ionic liquid (c).

We also evaluated the morphology of MWNT ion liquid by TEM, as shown in Figure 1. The diameter of the purified MWNT was about 40 nm, as shown in Figure 1(a); after RIGP, the diameter increased to 60 nm for the poly(GMA)-g-MWNT, as shown in Figure 1(b). When we introduced 1-butylimidazole bromide to an epoxy group of the grafted poly(GMA), the diameter increased to 80 nm, as shown in Figure 1(c). On the other hand, the morphology of the poly(GMA)-g-MWNT, as shown in Figure 1(b), was shown to be of tubular-type. The reason for the tubular-type morphology was considered as follows: in order to do this, we used as a vinyl monomer GMA, which is composed of hydrophilic >C=O (carbonyl group) and –C(O)-C- (epoxy group) sites and a hydrophobic vinyl group site. The vinyl group of the GMA comes to the surface of the MWNT because of a hydrophobic-hydrophobic interaction, while the carbonyl and epoxy group of monomer comes to the surface in an aqueous solution because of a hydrophilic-hydrophilic interaction.

When irradiated by γ-rays, the radical polymerization of the GMA occurs on the surface of the MWNTs. Hence, we successfully obtained the tubular-type functionalized MWNT as a one-step reaction. Thus, we could easily immobilize the enzyme to the functional group of the MWNT surface by physical adsorption, and we also used the MWNT as an electron transfer material in order to increase biosensor sensitivity.

**Figure 2.**
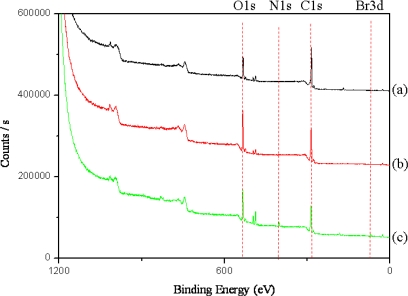
XPS survey scan spectra of pure MWNT (a), poly(GMA)-g-MWNT (b) and MWNT ionic liquid (c).

We also confirmed the successful synthesis of MWNT ion liquid via XPS spectroscopy analysis. Figure 2 shows a XPS survey scan spectra of pure MWNT (a), PGMA-g-MWNT (b) and MWNT ion liquid (c). There were significant changes after the introduction of butyl imidazolium bromide onto poly(GMA)-g-MWNT in the XPS data. The characteristic Br 3d peak at 70 eV and N 1s peak at 399 eV appeared after introduction of butyl imidazolium bromide onto poly(GMA)-g-MWNT. This illustrates that the MWNT ionic liquid was successfully prepared by the RIGP method.

**Figure 3.**
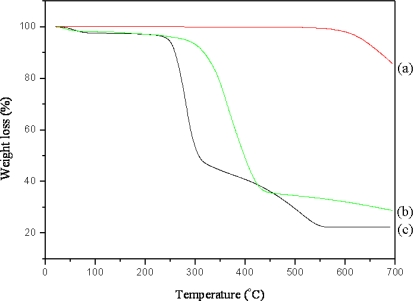
TGA curves of the purified MWNT (a), poly(GMA)-g-MWNT (b) and MWNT ionic liquid (c).

Figure 3 shows the TGA curves of the purified MWNT (a), poly(GMA)-g-MWNT and MWNT ionic liquid (c) prepared by RIGP. As shown in Figure 3, the 1^st^ weight loss (%) from 50 °C to 250 °C for poly(GMA)-g-MWNT and MWNT ion liquid appeared on account of moisture because of the hydrophilic properties of the grafted poly(GMA). The 2^nd^ weight loss appeared in a range of 250–600 °C due to the grafted poly(GMA) weight loss. As a result, the graft yield was approximately 40 % after RIGP of the GMA monomer. From these results, we confirmed the successful preparation of poly(GMA)-g-MWNT. However, 20% of MWNT remained after introduction of the imidazolium cations, as shown in Figure 3–c. As a result, it can be concluded that we were successful in the synthesis of the MWNT ionic liquid via RIGP.

### 3.2. Optimization of the Prepared MWNT Ion Liquid Electrode

**Figure 4.**
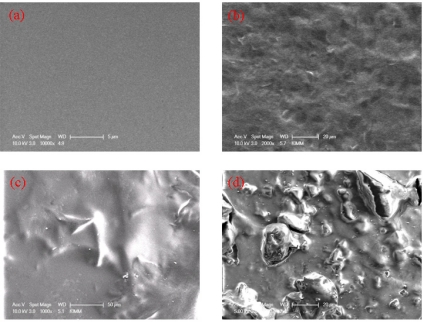
SEM images of ITO glass (a), chitosan-ITO glass (b), tyrosinase-chitosan-ITO glass (c) and MWNT ionic liquid electrode (d).

To improve the sensitivity of MWNT ion liquid toward tyrosinase, we introduced imidazolium cations to an epoxy group of poly(GMA)-g-MWNT prepared by the RIGP method. Subsequently, we fabricated a MWNT ionic liquid electrode by the hand-casting method (see Experimental section). Figure 4 reveals SEM images of the MWNT ion liquid electrode which include: ITO glass (a), ITO/chitosan (b), ITO/chitosan-tyrosinase (c), and a tyrosinase-immobilized MWNT ionic liquid electrode (d). Upon examination of the SEM images, the surface of ITO appears flat [Figure 3(a)], whereas the film of chitosan as a binder appeared as an amorphous flat form that resembles a typical polymer form, as shown in Figure 4(b). In contrast, the composite film with tyrosinase and chitosan appears as an irregular polymer form, as shown in Figure 4(c). In the MWNT ionic liquid electrode surface, the morphology of composite film changes and looks like irregular mountain patterns, as shown in Figure 4(d). As a result, we conclude that we successfully fabricated the MWNT ionic liquid electrode. The tyrosinase with plus charge strongly interacted with the anion charge of the MWNT ionic liquid. As a result, the irregular mountain morphology appeared, as shown in Figure 4(d).

**Figure 5.**
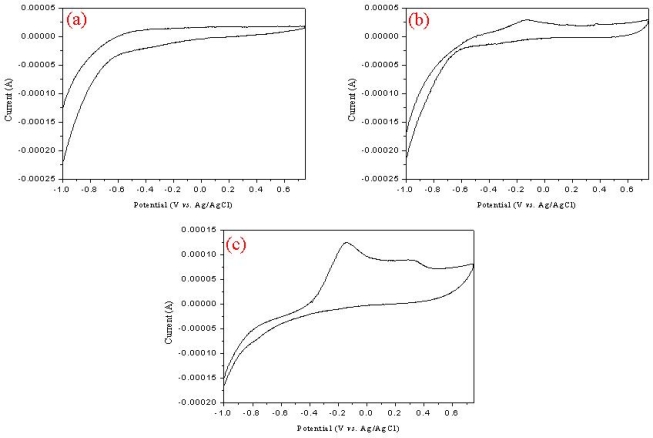
Cyclic voltammograms of phenol on the pure MWNT electrode with tyrosinase (a), chitosan electrode with tyrosinase (b), and MWNT ion liquid electrode with tyrosinase (c) in 2.0 mL phosphate buffer solution (pH = 7.0).

To determine the sensitivity of the MWNT ionic liquid electrode biosensor, we fabricated a tyrosinase-immobilized biosensor with or without the MWNT ionic liquid. Figure 5 shows the cyclic voltammograms of 0.5 mM phenol in 50 mM phosphate buffer solution using a pure MWNT electrode with tyrosinase (a), chitosan electrode with tyrosinase (b), and MWNT ion liquid electrode with tyrosinase (c). The potential is scanned between −1.0 and 0.75 versus Ag/AgCl at a scan rate of 100 mVs^−1^. The high oxidation peak at −0.2V and a low reduction peak at 0.2V appeared on the MWNT ion liquid electrode as shown in Figure 5(c). This peak indicates the production of catechol or *o*-quinone from the enzymatic reaction in phosphate solution. There were no oxidation peaks on other biosensors, as shown in Figure 5(a), in spite of the high concentrations used. On the other hand, a weak oxidation peak was observed at −0.2V on the chitosan electrode with tyrosinase in Figure 5(b). In short, tyrosinase is a copper-containing monooxygenase enzyme that catalyses the conversion of phenolic substrates to catechol and then *o*-quinones as follows:

(1) $$\text{phenol}+\text{tyrosinase}\;\left(O_{2}\right)\to \text{catechol}$$

(2) $$\text{catechol}+\text{tyrosinase}\;\left(O_{2}\right)\to o\text{-quinone}+H_{2}O$$

(3) $$o\text{-quinone}+H^{+}+{2e}^{-}\to \text{catechol}$$

**Figure 6.**
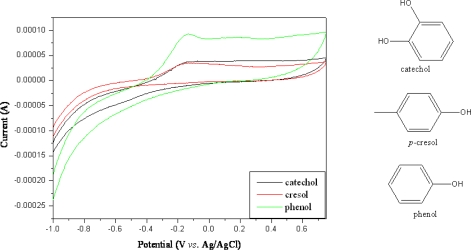
Cyclic voltammograms of MWNT ionic liquid electrode based on tyrosinase in 2.0 mL phosphate buffer solution (pH = 7.0) with 0.04 mM catechol, 0.04 mM *p*-cresol, 0.04 mM phenol.

Selectivity is a very important factor in biosensors, and three compounds, phenol, *p*-cresol, and catechol, were detected by the MWNT ionic liquid electrode based on tyrosinase in a 50 mM phosphate buffer solution (pH = 7.0), as shown in Figure 6. In the *p*-cresol and catecohol solution, there was a weak redox peak for the MWNT ionic liquid electrode based on tyrosinase for this experiment. Serra *et al*. [21] reported that different sensitivity was observed for phenolic compounds with different substitution patterns in an enzymatic reaction. The *meta*-position phenol (*m*-cresol) appeared to have a high response sensitivity as compared to *ortho*- and *para*-position phenols in an enzymatic reaction due to its high affinity for tyrosinase. As shown in Figure 6, a high response peak appeared for phenol as compared to *p*-cresol and catecohol on account of its enzymatic reaction. Tsai *et al*. [22] reported that *p*-catechol gave a high response sensitivity as compared to dopamine and ephinephrine. However, a low sensitivity response for catecohol appeared with the MWNT ionic liquid electrode based on tyrosinase.

**Figure 7.**
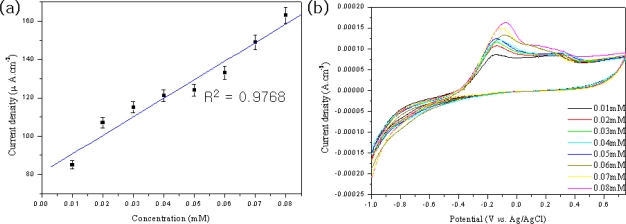
Cyclic voltammograms of MWNT ion liquid electrode based on tyrosinase in 2.0 mL phosphate buffer solution (pH = 7.0) containing 0.01∼0.08 mM phenol (a), (b).

The electrochemical biosensing of phenols was performed under optimal experimental conditions. Figure 7 shows the cyclic voltammograms of phenols on the MWNT ionic liquid electrode based on tyrosinase in a 50 mM phosphate buffer solution (pH = 7.0) as a function of phenol concentration. The detection response range for phenol was from 0.01 to 0.08 mM concentration, as shown in Figure 7. The sensitivity and apparent Michaelies-Menten constant (K_M_^app^) of the MWNT ion liquid based on tyrosinase were 2.925 A (mol·L^−1^) ^−1^ and (0.041) mmol·L^−1^, respectively. The K_M_^app^ values were lower than those reported for the free enzymes in solution, which was estimated to be 700 μmol·L^−1^ as a substrate [23]. Such low K_M_^app^ values may be attributed to the high concentration of tyrosinase on the biosensor. In addition, the embedded functionalized MWNT in a composite film may have increased the access of substrate molecules to enzyme catalytic sites.

**Figure 8.**
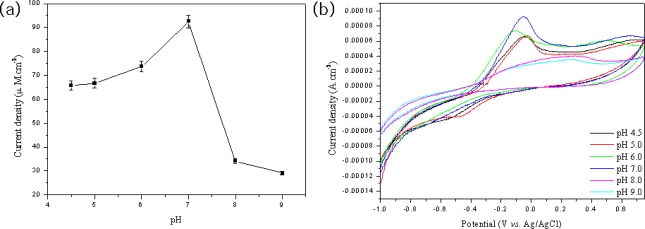
Response sensitivity of the MWNT ion liquid electrode based on tyrosinase as function of pH value for 0.04 mM phenol in 2 mL phosphate buffer (a), (b).

Another parameter affecting the sensing efficiency of the MWNT ionic liquid electrode based on tyrosinase is the pH of the supporting electrolyte. Figure 8 presents cyclic voltammograms of 0.04 mM phenol on the MWNT ionic liquid electrode based on tyrosinase in 50 mM phosphate buffer solution as a function of pH. The sensing efficiency increased with increasing pH levels from 4.0 to 7.0, and then rapidly decreased with increasing pH levels, as shown in Figure 8. This means that at low pH levels, the sensing efficiency of biosensors is due to enzyme activity.

**Figure 9.**
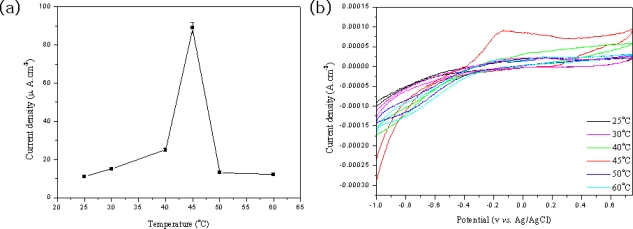
Response sensitivity of the MWNT ion liquid electrode based on tyrosinase as a function of temperature for 0.04 mM phenol in 2.0 mL phosphate buffer (a), (b).

The effect of temperature on the amperometric response of phenol was also studied and is shown in Figure 9. Prior to cyclic voltammetry detection, the MWNT ionic liquid electrode based on tyrosinase was immersed into a buffer solution at a given temperature for 10 minutes, and then the CV was recorded. As shown in Figure 9, the response sensitivity increased with increasing temperatures from 20 to 45 °C, and then rapidly decreased when temperatures were increased. Maximum sensitivity was attained at 45 °C. At high temperatures, the response sensitivity of the MWNT ionic liquid electrode based on tyrosinase for phenol rapidly decreased due to partial breakdown of the immobilized tyrosinase. Although the response sensitivity of the biosensor was greatest at 45 °C, considering that most enzymes can be easily broken down at high temperatures and the practical application that requires a simple experimental procedure and the long lifetime of the biosensor, room temperature was used consistently throughout the study.

### 3.3. Total Amounts of Phenolics in Commercial Red Wines

**Figure 10.**
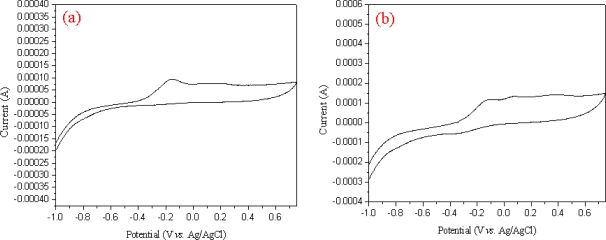

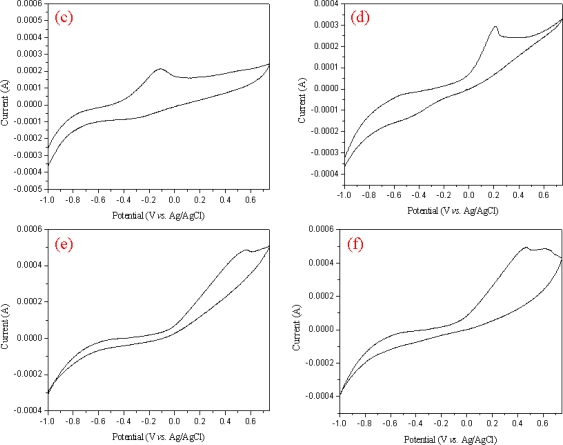
Cyclic voltammograms of MWNT ion liquid electrode based on tyrosinase in 2.0 mL phosphate buffer solution (pH = 7.0) containing 8 mL Chateau Mani-dry (a), Chateau Mani-sweet (b), Chateau Mani-nouveau (c) and in 2.0 mL Chateau Mani-dry (d), Chateau Mani-sweet (e), Chateau Mani-nouveau (f) as electrolyte.

Three red wines: Brand Chateau Mani-dry, Chateau Mani-sweet, Chateau Mani-nouveau, made in Korea, were used in the analysis of phenolic compound amounts in wines. Figure 10 shows cyclic voltammograms of the MWNT ionic liquid electrode based on tyrosinase in a 2.0 mL phosphate buffer solution (pH = 7.0) containing 8.0 μL Chateau Mani-dry (a), 8.0 μL Chateau Mani-sweet (b), 8.0 μL Chateau Mani-nouveau (c), respectively, and in 2.0 mL Chateau Mani-dry (d), 2.0 mL Chateau Mani-sweet (e), 2.0 mL Chateau Mani-nouveau (f) plain red wine. As shown in Figure 10(a–c), the oxidation peak of red wine in a phosphate buffer using the MWNT ionic liquid electrode appeared at −0.2 V, while the phenol peak in plain red wine without a phosphate buffer shifted to a plus potential in Figure 10(d–f).

Table 2 summarizes the total phenolics in a phosphate buffer and plain red wine on the MWNT ionic liquid electrode based on tyrosinase at room temperature. Table 2 presents the amount of phenolics in commercial red wines in the range of 383.5–3,087 mg/L in a phosphate buffer solution.

## 4. Conclusions

In this study, we fabricated an immobilized tyrosinase biosensor based on a MWNT ionic liquid prepared by radiation-induced graft polymerization. The sensing range of the tyrosinase-immobilized biosensor based on MWNT ionic liquid for phenol was from 0.01 to 0.08 mM. The tyrosinase-immobilized biosensor based in the MWNT ionic liquid was optimimized for pH, temperature, and other phenolic compounds. The prepared biosensor was used to determine phenolics in commercial red wines. As a result, the amount of phenolics in commercial red wines have been determined to be in the range of 383.5–3,087 mg/L in a phosphate buffer solution. This range was calculated from a calibration curve of phenols on a tyrosinase-immobilized biosensor based on a MWNT ionic liquid electrode. The high amounts of phenolic compounds in Brand Chateau Mani-nouveau appear to be responsible for the bitter taste of this red wine.

## Figures and Tables

**Table 1.** Elemental analysis of the prepared MWNT ion liquids.

**Sample**	**N (%)**	**C (%)**	**H (%)**	**O (%)**
Pure MWNT		76.14	0.74	11.81
poly(GMA)-g-MWNT		66.78	4.80	22.25
MWNT ionic liquid	5.35	55.79	4.63	18.58

**Table 2.** Total phenolic concentration determined by the MWNT ionic liquid electrode based on tyrosinase for commercial red wines.

**Commercial red wine (Korea)**	**Current density**	**Phenolics ^a)^**	**Phenolics ^b)^**
Chateau Mani-dry (Korea)	9.7×10^−5^ A	383.5 mg/L	20.33 mg/L
Chateau Mani-sweet (Korea)	1.2×10^−4^ A	872.9 mg/L	38.02 mg/L
Chateau Mani-nouveau (Korea)	2.1×10^−4^ A	3087 mg/L	39.01 mg/L

a) in phosphate buffer containing 8 mL commercial red wine.

b) in crude red wine of 2.0 mL.
